# Examining maladaptive eating behaviors and psychological difficulties among women with compulsive eating and obesity: a latent profile analysis

**DOI:** 10.1186/s40337-025-01193-2

**Published:** 2025-02-19

**Authors:** Charlie Maltais-Lévesque, Maxime Legendre, Catherine Bégin

**Affiliations:** 1 School of Psychology, Laval University, 2325 rue des Bibliothèques, Quebec City, QC G1V 0A6 Canada; 2 Centre d’Expertise Poids, Image et Alimentation (CEPIA), Institute of Nutrition and Functional Foods, 2440 Bd Hochelaga, Quebec City, QC G1V 0A6 Canada

**Keywords:** Compulsive eating, Classification, Eating behaviors, Obesity, Psychotherapy

## Abstract

**Background:**

Maladaptive eating behaviors are central to weight gain and are influenced by various psychological determinants associated with obesity. While some studies have established profiles based on different maladaptive eating behaviors using medical samples of individuals with obesity, no studies have focused on all patients with overweight or obesity seeking psychological help for compulsive eating. The objective was to identify eating behaviors profiles using maladaptive eating behaviors (disinhibition, susceptibility to hunger, and restraint) among women patients with compulsive eating and overweight or obesity, and to compare those profiles with each other on socio-demographic, clinical, psychological, and eating-related variables.

**Methods:**

One hundred and eighty-eight women patients with overweight or obesity (BMI ≥ 25 kg/m^2^) completed online questionnaires and participated in an eating disorders diagnosis interview. Profiles were created using latent profile analysis and compared with a MANOVA with Tukey adjusted post-hoc comparisons and a chi-square analysis.

**Results:**

Three profiles emerged from the latent profile analysis. The *Highly disinhibited* profile includes women with high scores for disinhibition and susceptibility to hunger and a low score of restraint. The *Moderate sensitivity to eating cues* profile includes women with moderate scores of disinhibition, susceptibility to hunger, and restraint. The *Perceived control over food* profile includes women with the highest restraint score and the lowest levels of disinhibition and susceptibility to hunger. Group comparisons showed significant differences between profiles. The *Highly disinhibited* profile included women with higher levels of depressive symptoms and impulsivity as well as lower scores of self-directedness and cooperation. This profile also showed the highest level of binge eating, food craving, and food addiction symptoms. The *Moderate sensitivity to eating cues* profile showed more body esteem and greater cooperation. The *Perceived control over food* profile had less impulsivity and depressive symptoms as well as a higher level of self-directedness.

**Conclusion:**

These results highlighted mechanisms that seem to prevail in different profiles of patients with compulsive eating which offer intervention targets that should be prioritized when offering psychotherapeutic treatment.

## Abstract (plain language summary)

Eating behaviors are important contributing factors to weight gain and are influenced by many psychological characteristics. Classification of patients based on eating behaviors is an efficient way to better understand how patients with compulsive eating and obesity differ from one another and offer adapted treatment. In this study, three eating behaviors were used (disinhibition, susceptibility to hunger, and restraint) to establish profiles in a sample of 188 women patients with compulsive eating and overweight or obesity. Three profiles were identified, namely (1) *Highly disinhibited*, (2) *Moderate sensitivity to eating cues*, and (3) *Perceived control over food.* When compared with each other, these profiles showed differences in impulsivity, body esteem, depressive symptoms, personality traits (self-directedness and cooperation), binge eating, food craving, and food addiction. Future research could pair these profiles with specific interventions to improve treatment success.

## Introduction

Obesity is a major health problem around the world and Western countries are among the most affected [1]. In Canada, nearly 30% of the adult population reported a body mass index (BMI) over 30 kg/m^2^ and therefore reach the obesity threshold [2]. The worldwide prevalence of obesity and its related consequences (i.e., diabetes, metabolic syndrome, depressive symptoms, low quality of life, and weight stigmatization) have encouraged researchers to better understand its contributing factors [3–7]. Several genetic, neurobiological, environmental, and individual factors have been identified as important factors related to obesity and weight gain [8]. Among all these factors, maladaptive eating behaviors have received considerable attention as a potential intervention target by health professionals [9]. Maladaptive eating behaviors refer to eating patterns that would have long-term physical or psychological consequences, whether characterized by excessive restriction (restraint eating) or an inability to inhibit food intake (compulsive eating) [10]. These behaviors can be quite heterogeneous, with some patients exhibiting only compulsive eating or restraint eating, while others display both compulsive and restraint behaviors, such as binge eating episodes triggered by calorie counting [11, 12].

Up until now, researchers have attempted to identify, based on maladaptive eating behaviors, profiles associated with obesity to create more homogeneous subgroups of individuals. Initial profiling efforts were made by Claes et al. [13] and replicated by Müller et al. [14] who characterized 102 and 156 patients respectively with severe obesity awaiting bariatric surgery. Although their profiles were based on personality traits, the authors took care to distinguish them using eating behaviors. More precisely, they found two profiles and compared them with each other on eating-related variables (e.g., external eating, restraint, and weight concerns), and psychological variables such as emotion regulation and impulsivity. The profile named “emotionally dysregulated/undercontrolled” showed that the presence of more negative personality traits (i.e., high neuroticism, low agreeableness, and low conscientiousness) was associated with more maladaptive eating behaviors as well as higher levels of impulsivity, less adaptive emotion regulation strategies, and greater body dissatisfaction compared to the profile named “resilient/high functioning”. These results highlighted the importance to consider simultaneously psychological traits and emotion regulation in conjunction with maladaptive eating behaviors.

More recently, three studies have lingered to characterize patients based on eating behaviors and eating-related variables. Heerman et al. [15] characterized people with normal weight, overweight, or obesity (*n* = 9,977) mostly consulting for primary care using maladaptive eating behaviors (i.e., emotional, excessive, and impulsive eating) as well as eating patterns (i.e., eating healthy and unhealthy food frequencies, breakfast and snacking frequencies, and overall diet quality). They found four profiles: (1) healthy, (2) healthy with maladaptive eating behaviors, (3) unhealthy and (4) unhealthy with maladaptive eating behaviors. Early on, the authors highlighted that profile named “unhealthy with maladaptive eating behaviors” included patients with the highest scores on overeating, emotional eating and impulsive eating as well as on the unhealthy diet scale (i.e., consumption of highly processed food) and the highest BMI among the four profiles. Their results show that maladaptive eating behaviors were key variables associated with the risk of obesity, underlining the importance of considering maladaptive eating behaviors when profiling people with higher BMI.

Romain et al. [16] characterized 126 patients with severe obesity consulting for nonsurgical weight loss treatment based on maladaptive eating behaviors (i.e., uncontrolled eating, cognitive restraint, emotional eating, and external eating) and several additional obesity-related variables such as physical activity, sedentary time, tobacco use, depressive symptoms, health-related quality of life, waist circumference, and socio-demographic informations. The authors found three profiles. The first profile named “struggling with food” was characterized by the highest scores on disinhibited eating measures, the second profile named “low loss of eating control” had the lowest score on the uncontrolled eating subscale while the third profile named “pleasure from eating” showed the highest score on the comfort with food subscale. Despite the inclusion of many relevant variables associated with physical health such as physical activity, sedentary time, tobacco use, and health-related quality of life, maladaptive eating behaviors were the only variables that help differentiate their profiles. Although this study shows that maladapting eating behaviors emerge as a determining factor, it would have been interesting to compare the profiles based on psychological variables, which was not done in this study.

A recent study specifically examined profiles based on eating behaviors and psychological symptoms in a clinical population with food addiction, a construct associated with binge eating and obesity. Jiménez-Murcia et al. [17] used an initial sample of 395 patients assessed for an eating disorder (ED) from which they retained 234 women with self-reported food addiction to identify profiles that could be differentiated in terms of eating behaviors and symptoms (i.e., drive for thinness, bulimic symptoms, awareness of internal cues), personality traits and psychiatric symptoms. They found three profiles: (1) dysfunctional, (2) moderate, and (3) functional. The profiles they identified mostly reflect a continuum of severity for food addiction. Results show that the dysfunctional profile was characterized by the highest scores on eating behaviors like drive for thinness and impulsive eating as well as on psychological variables such as depressive symptoms. Women in this profile also had the lowest scores on self-directedness and cooperation personality traits. The moderate profile was characterized by patients with the longest duration of eating problems, while the functional profile included patients with more adaptive scores on clinical variables and higher scores on personality traits such as self-directedness and cooperativeness. While food addiction is a relevant construct in understanding obesity, many patients with obesity do not necessarily meet criteria for food addiction, yet still exhibit a pathological relationship with food. Moreover, the identification of a functional profile among a population with food addiction may be questionable, as food addiction is widely recognized as a marker of severity in maladaptive eating behaviors [18, 19].

To date, efforts have been made to establish maladaptive eating behaviors profiles among patients with obesity, mainly seeking weight loss treatment or with food addiction and ED. Unfortunately, none of these samples represent the full range of patients seen in primary care for compulsive eating. Including all patients seeking help for compulsive eating and weight concerns in profile studies would provide a better understanding of their characteristics, enabling the identification of intervention strategies tailored to their distinct needs and the specific concerns that led them to seek help. To this end, the use of three observable maladaptive eating behaviors allows for a quick and simple classification that could efficiently inform treatment. Hence, the first aim of this study was to characterize patients seeking help for compulsive eating using disinhibition, susceptibility to hunger, and restraint. The second aim was to compare the maladaptive eating behaviors profiles with each other on clinically relevant psychological and eating-related characteristics such as impulsivity, emotion regulation strategies, body esteem, depressive symptoms, personality traits, binge eating, food craving, and food addiction, as well as socio-demographic and clinical variables. It was hypothesized that at least three profiles would be found, one characterized by high levels of compulsive eating, one with more pronounced restraint eating and the other with a mix presentation of compulsive and restraint eating. We hypothesized that the three profiles would differ on psychological variables, and specifically that the compulsive one would show a more dysregulated presentation.

## Method

### Participants

The original sample consisted of 211 men and women who sought psychological help for compulsive eating. Eligibility criteria included (1) to be at least 18 years old, (2) to have a BMI over 25 kg/m^2^, and (3) to seek psychological help for compulsive eating. To reduce sample heterogeneity, patients with bulimia (*n* = 3) and atypical anorexia (*n* = 2) as well as men (*n* = 18) were excluded. Therefore, the final sample was composed of 188 women with a mean age of 44.28 (SD = 12.58) and a mean BMI of 37.75 (SD = 7.45). For ED diagnosis, 45 patients had binge eating disorder, 15 patients had otherwise specified feeding and eating disorder (OSFED), 49 patients had eating disorder not otherwise specified (EDNOS), and 57 patients did not meet the criteria for any ED while still reporting compulsive eating behaviors. Socio-demographic and clinical characteristics for this sample are presented in Table 1.

**Table 1:** Socio-demographic and clinical characteristics

Variables	%
*Socio-demographic*	
Age	
18 to 34 years	26.06
35 to 49 years	39.36
50 to 64 years	28.73
65 years and older	5.85
Ethnicity	
Caucasian	97.87
Latino	1.60
Did not answer	0.53
Occupation	
Studying	15.43
Working	73.40
Other	11.17
Education	
High school	14.89
College	29.26
University	55.85
Income	
Less than 40 000$ CAD	19.68
40 000$ to 80 000$ CAD	30.32
More than 80 000$ CAD	38.30
Did not answer	11.70
Family status	
Married/In a relationship	57.98
Single/Widow	27.66
Divorced/Separated	13.83
Did not answer	0.53
*Clinical*	
BMI	
Overweight	28.19
Obesity	71.81
Eating disorder diagnosis	
Binge eating disorder	23.94
Otherwise specified feeding and eating disorder (OSFED)	7.98
Eating disorder not otherwise specified (EDNOS)	26.06
No eating disorder	30.32
Not available	11.70

### Procedure

Participants were recruited at the Centre d’Expertise Poids, Image et Alimentation (CEPIA), a multidisciplinary clinic specializing in ED treatment. They were invited to participate in a clinical interview including an ED diagnosis checklist based on the fifth edition of the Diagnostic and Statistical Manual of Mental Disorders (DSM-5) [20] conducted by psychologists or graduate students under supervision. They also completed questionnaires on LimeSurvey. The questionnaires were completed within seven days of the clinical interview. The checklist uses the DSM-5 criteria for each ED (i.e., AN, BN, BED, OSFED, and EDNOS) and allows the clinician to verify the presence or absence of an ED diagnosis as well as the severity of the ED (e.g., binge frequency). The recruitment period began in April 2019 and ended in March 2023. Each participant provided inform consent. This study was approved by the Comité d’éthique de la recherche avec des êtres humains de l’Université Laval (CÉRUL).

### Measures

#### Classification variables

##### Disinhibition, susceptibility to hunger, and restraint

The three subscales of the French version of the Three Factor Eating Questionnaire (TFEQ) were used to measure eating behaviors, namely disinhibition, susceptibility to hunger, and restraint [21, 22]. The disinhibition subscale consists of 16 items and measures episodes of loss of control over food consumption (e.g., “Sometimes when I start eating, I just can’t seem to stop”). The susceptibility to hunger subscale consists of 14 items and focuses on subjective feeling of hunger influenced by internal (emotions, cravings) and external cues (e.g., “Sometimes what I eat seems so good that I continue to eat even though I’m not hungry anymore”). The restraint subscale is composed of 21 items and measures eating restriction that aims to influence one’s weight or body shape (e.g., “During meals, I purposely restrict myself so I don’t gain weight”). This questionnaire is divided into two parts, the first consisting of questions that are answered with “True” or “False” and the second consisting of items answered with a Likert scale ranging from 1 (“Rarely”) to 4 (“Always”), with a higher score corresponding to a higher level of disinhibition, susceptibility to hunger, and restraint. All subscales of this questionnaire show good Cronbach’s alpha, with scores ranging from 0.79 to 0.93 [22]. The internal consistency of the TFEQ and each subscale for the present sample were adequate, Cronbach’s alpha ranging from 0.61 to 0.82.

#### *Comparison variables*

##### Socio-demographic and clinical variables

Age, sex, ethnicity, occupation, education, income, family status, BMI, and ED diagnosis were collected.

##### Impulsivity

 The 20-item short version and validated in French of the UPPS Impulsive Behavior Scale was used [23–25]. It includes five subscales composed of 4 items respectively. Each item describes ways of acting or thinking (e.g., “I usually make up my mind after careful consideration”). For this study, the global score of impulsivity was used. Participants are asked to indicate their level of agreement with the item using a Likert scale ranging from 1 (“Strongly agree”) to 4 (“Strongly disagree”). The total score is obtained by summing the items, a higher score indicating the presence of greater impulsivity. The subscales had acceptable internal consistency with Cronbach’s alphas ranging from 0.77 to 0.83 [24]. The internal consistency for this sample was adequate (α = 0.83).

##### Emotion regulation

The French version of the Cognitive Emotion Regulation Questionnaire (CERQ), which contains 36 items, present various cognitive strategies used to regulate emotions during negative or unpleasant events [26, 27]. The items are separated equally into two subscales, one focusing on adaptive emotion regulation strategies and the other on non-adaptive emotion regulation strategies. Participants are asked to indicate the frequency they used these strategies by responding on a Likert scale ranging from 1 (“Almost never”) to 5 (“Almost always”). The score for each subscale is obtained by summing the items, with a higher score indicating a higher frequency of use of this type of regulation strategy. Cronbach alphas for this questionnaire range from 0.75 to 0.87 and test-retest correlations range from 0.48 to 0.65, indicating acceptable psychometric characteristics [28]. The internal consistency of the CERQ and each subscale for the present sample were adequate, Cronbach’s alpha ranging from 0.87 to 0.91.

##### Body esteem

The French version of the Body-Esteem Scale for Adolescents and Adults (BESAA) was used to measure body esteem [29, 30]. It consists of three subscales addressing general feelings related to appearance satisfaction (e.g., “I like what I see when I look in the mirror”), weight satisfaction (e.g., “I like my weight”), and attribution of positive evaluations of one’s body and appearance in relation to others (e.g., “I am as beautiful as most people”) [30]. The 23-item scale is answered on a Likert scale ranging from 0 (“Never”) to 4 (“Always”) and indicates the individual’s level of agreement with the items. The score for each dimension represents the means of the answers, with a higher score indicating higher body esteem. Cronbach’s alphas for all three subscales range from 0.91 to 0.93, signifying that the instrument has good internal consistency [31]. The internal consistency of the BESAA and each subscale for this sample were adequate, Cronbach’s alpha ranging from 0.65 to 0.87.

##### Depressive symptoms

The presence and severity of depressive symptoms in the participants was assess using the French version of the Beck Depression Inventory-II (BDI-II) [32, 33]. The BDI-II is a 21-item self-report questionnaire. Items are answered by a Likert scale ranging from 0 (“No suffering”) to 3 (“Intense suffering”). The total score is calculated by summing the items: a score between 0 and 13 indicates minimal symptom severity, a score between 14 and 19 indicates mild depression, a score between 20 and 28 indicates moderate depression, and a score between 29 and 63 indicates severe depression. For the sample of this study, the Cronbach’s alpha of 0.91 suggests the internal consistency is excellent.

##### Personality traits

The French version of the Temperament and Character Inventory (TCI-125) was used to observe personality traits [34, 35]. This 125-item self-report questionnaire is a shortened version of the 226-item TCI. It was developed to assess the temperament and character of individuals along seven personality dimensions. Of these seven dimensions, four are temperament dimensions (novelty seeking, harm avoidance, reward dependence, and persistence). The three character dimensions are: self-directedness, cooperativeness, and transcendence. The TCI demonstrates good internal validity, ranging from 0.76 to 0.89 [35]. The internal consistency of the TCI and each subscale for this sample were adequate, Cronbach’s alpha ranging from 0.54 to 0.86.

##### Binge eating

The Binge Eating Scale (BES) was used to measure behaviors correlated to binge eating episodes among the participants [36]. The 16-item scale is made of statements with four response choices. Participants are encouraged to choose the option that suits their situation best. Each item has his own level of severity varying from 0 to 3. The total score can be summed between 0 and 46, a higher score meaning a more severe binge eating pathology. The internal consistency of the BES for this sample was good, with a Cronbach’s alpha of 0.86.

##### Food craving

The Food Craving Questionnaire – Trait, Reduced (FCQ-T-r) was used to assess food craving [37]. The FCQ-T-r is the reduced version of the FCQ-T. It is a self-reported questionnaire composed of 15-item evaluating the global presence of food craving in which the participants must agree with the answer best representing their reality using a Likert scale ranging from 0 (“Never”) to 5 (“Always”). Items can be summed with a global score ranging between 0 and 75. The internal consistency of the FCQ-T-r for this sample was excellent, with a Cronbach’s alpha of 0.93.

##### Food addiction

Food addiction symptoms were assessed using the Yale Food Addiction Scale Version 2.0. (YFAS) [38]. The YFAS is composed of 35-item and measure food addiction based on the 11 DSM-5 criteria for substance-used disorders (APA, 2013). A Likert Scale ranging from 0 (“Never”) to 5 (“Everyday”) is used to answer the items. For this study, the food addiction severity was used to examine the symptoms among the participants. A sum of the endorsed criteria from 0 to 11 was used to compare food addiction between the profiles. The internal consistency of the YFAS for this sample was excellent, with a Cronbach’s alpha of 0.92.

### Data analysis plan

For the first objective, profiles were identified using Mplus software. Latent profile analysis (LPA) was performed with total scores of the disinhibition, susceptibility to hunger, and restraint subscales. This analysis was chosen because it allows for the creation of homogeneous groups across a heterogeneous sample [39]. The degree of entropy, ranging from 0 to 1, was used to test the quality of model classification, with a higher-level meaning that participants are classified into a model that is more appropriate for them [40]. Log-Likelihood, Bayesian Information Criterion (BIC) and Aikaike Information Criterion (AIC) indexes were used to help select the best model from those proposed by the LPA, with a value approaching 0 for these indexes indicating models closer to reality [40–42]. Adjusted Lo-Mendell-Rubin test (Adjusted LMR) and Bootstrapped likelihood ratio test (BLRT) were also used to choose the solution, significant (*p* <.05) LMR and BLRT indicating the model can be retained [43–45].

For the second objective, IBM SPSS (version 29) was used to observe how the profiles differ from each other. A MANOVA with Tukey adjusted post-hoc comparisons was performed to observe differences between profiles for the classification variables as well as for the comparison variables. Moreover, a chi-square analysis was used to compare profiles on socio-demographic and clinical variables (i.e., age, sex, ethnicity, occupation, education, income, family status, BMI, and ED diagnosis).

## Results

### Identification of eating behaviors profiles

Models ranging from 1 to 4 profiles were tested using LPA. The Log-Likelihood detected the biggest drop between the one- and two-profiles groups and showed the second biggest drop between the two- and three-profiles groups. The AIC and BIC fit statistics were the lowest for the three-profiles model. The LMR and BLRT were significant for the two- and three-profiles solution and the entropy was adequate for both models. As the three-profile solution was supported by all the fit indices, we made the final decision to conserve three profiles. The results are presented in Table 2.

**Table 2:** Fit statistics for latent profile analysis

Number of profiles	LL	AIC	BIC	LMR (*p*)	BLRT (*p*)	Entropy
1	-1496.59	3005.17	3024.59	-	-	-
2	-1471.09	2962.18	2994.54	0.00	0.00	0.69
**3**	**-1459.37**	**2946.75**	**2992.06**	**0.00**	**0.00**	**0.79**
4	-1455.46	2946.94	3005.17	0.07	0.38	0.80

*LL* Log-Likelihood, *AIC* Aikaike Information Criterion, *BIC* Bayesian Information Criterion, *LMR* Adjusted Lo-Mendell-Rubin test, *BLRT* Bootstrapped likelihood ratio test

Boldface type indicates the selected model

### Profiles differences

#### Classification variables

Profile 1 named “*Highly disinhibited*” had the highest scores on disinhibition and susceptibility to hunger and the lowest on restraint. Profile 2 named “*Moderate sensitivity to eating cues*” included patients with high scores on all eating behaviors subscales without being the most severe in the sample. Finally, profile 3 named “*Perceived control over food*” had the highest restraint score and the lowest disinhibition and susceptibility to hunger scores among the three profiles. Results are presented in Table 3.

**Table 3:** Identification of eating behaviors profiles

Variables	Eating behaviors profiles			
	Highly disinhibited (*n* = 73)	Moderate sensitivity to eating cues (*n* = 76)	Perceived control over food (*n* = 39)	*F*
Disinhibition	12.42 (1.94)^a^	11.12 (2.10)^b^	9.21 (2.79)^c^	27.36*
Habitual	3.89 (1.24)^a^	3.18 (1.33)^b^	2.64 (1.60)^b^	11.65*
Emotional	2.79 (0.53)^a^	2.53 (0.84)^a, b^	2.21 (1.15)^b^	6.76*
Situational	4.26 (1.03)^a^	3.66 (1.16)^b^	2.74 (1.53)^c^	20.38*
Susceptibility to hunger	11.53 (1.45)^a^	6.55 (1.42)^b^	2.26 (1.16)^c^	607.35*
Internal	5.08 (1.00)^a^	2.47 (1.24)^b^	0.54 (0.79)^c^	251.99*
External	4.84 (0.83)^a^	2.93 (1.17)^b^	1.05 (0.83)^c^	196.54*
Restraint	7.26 (4.26)^a^	7.93 (4.21)^a, b^	9.36 (4.52)^b^	3.05*
Flexible	3.11 (1.09)^a^	3.36 (2.07)^a^	3.93 (1.97)^a^	2.28
Rigid	2.68 (1.62)^a^	2.61 (1.63)^a^	2.97 (1.78)^a^	0.66

Means with different subscripts within a row are significantly different from one another (*p* <.05)

* *p* <.05

#### *Comparison variables*

In addition to eating behaviors, other variables were compared to better understand the differences between the profiles. Initially, profiles were compared on socio-demographic and clinical variables. Firstly, chi-square analysis showed no significant differences between profiles with respect to ethnicity (χ2 = 1.87, *p* =.76), occupation (χ2 = 3.08, *p* =.22), education (χ2 = 3.43, *p* =.49), income (χ2 = 9.41, *p* =.67), and family status (χ2 = 12.71, *p* =.12). ED diagnosis differed significantly between the profiles, *Highly disinhibited* and *Moderate sensitivity to eating cues* including significantly more people with binge eating disorder with 24 and 19 patients respectively compared to *Perceived control over food* with only 2 patients (χ2 = 18.88, *p* <.01). Secondly, a MANOVA with Tukey adjusted post-hoc comparisons was used to compare profiles based on age and BMI (see Table 4). We found a difference for age, with patients in the *Moderate sensitivity to eating cues* profile being significantly older than those in the *Highly disinhibited* profile. No difference was found for BMI between the profiles.

Differences in psychological variables were also observed (see Table 4). For impulsivity, *Highly disinhibited* showed significantly higher scores than *Perceived control over food*, which had the lowest impulsivity scores among the three profiles. Regarding adaptive and non-adaptive emotion regulation strategies, there was no difference between the profiles. Furthermore, body esteem differences were observed. The score on the body esteem related to appearance subscale was significantly higher for *Moderate sensitivity to eating cues* compared to *Highly disinhibited*. There was no significant difference between *Perceived control over food* and the two other profiles on the body esteem related to appearance subscale and no difference between the three profiles on the two other body esteem subscales (weight and attribution). For depressive symptoms, patients in *Highly disinhibited* showed significantly higher depressive symptoms compared to those in *Perceived control over food.* Differences between personality traits were also observed. Among all the traits, only self-directedness and cooperativeness showed significant results. *Highly disinhibited* showed significantly lower scores of self-directedness compared to *Moderate sensitivity to eating cues* and *Perceived control over food.* Patients in the *Moderate sensitivity to eating cues* profile also showed significantly lower scores on self-directedness compared to the *Perceived control over food* profile. Furthermore, *Moderate sensitivity to eating cues* showed a significant higher level of cooperativeness compared to *Highly disinhibited*, without being different from *Perceived control over food. Highly disinhibited* and *Perceived control over food* showed no significant difference.

Finally, differences between the three profiles regarding other eating-related variables completed the picture (see Table 4). For binge eating and food craving, *Highly disinhibited* showed the highest scores compared to the two other profiles. *Moderate sensitivity to eating cues* also showed higher scores for binge eating and food craving compared to the *Perceived control over food* profile, which had lower scores. For food addiction, *Highly disinhibited* showed significantly higher scores compared to *Moderate sensitivity to eating cues* and *Perceived control over food. Moderate sensitivity to eating cues and Perceived control over food* did not differ from each other.

**Table 4:** Means (and standard deviations) of socio-demographic, clinical, psychological and eating-related variables for each eating behaviors profile

Variables	Eating behaviors profiles			
	Highly disinhibited (*n* = 73)	Moderate sensitivity to eating cues (*n* = 76)	Perceived control over food (*n* = 39)	*F*
*Socio-demographic and clinical*				
Age	41.19 (13.86)^a^	46.28 (11.63)^b^	44.28 (12.58)^a, b^	3.70*
BMI	37.38 (5.72)^a^	38.34 (7.96)^a^	37.12 (8.87)^a^	0.32
*Psychological*				
Impulsivity	45.54 (7.74)^a^	44.12 (6.49)^a, b^	41.31 (7.94)^b^	3.99*
Adaptive emotion regulation strategies	58.08 (13.97)^a^	60.46 (11.01)^a^	59.33 (14.59)^a^	0.35
Non-adaptive emotion regulation strategies	42.14 (11.08)^a^	38.24 (9.21)^a^	36.72 (8.01)^a^	2.43
Body esteem – Appearance	0.75 (0.52)^a^	1.06 (0.71)^b^	0.89 (0.65)^a, b^	4.43*
Body esteem – Weight	0.46 (0.46)^a^	0.68 (0.63)^a^	0.54 (0.58)^a^	2.75
Body esteem – Attitude	1.44 (0.62)^a^	1.46 (0.60)^a^	1.48 (0.85)^a^	0.05
Depressive symptoms	18.55 (11.89)^a^	14.54 (11.06)^a, b^	14.00 (9.27)^b^	3.06*
Novelty Seeking	42.95 (17.99)^a^	42.30 (18.88)^a^	37.76 (18.91)^a^	1.06
Harm Avoidance	67.04 (24.95)^a^	58.47 (24.85)^a^	64.45 (23.40)^a^	2.34
Reward Dependence	73.77 (15.18)^a^	70.62 (14.99)^a^	72.13 (15.24)^a^	0.81
Persistence	70.14 (30.39)^a^	70.00 (27.81)^a^	73.16 (22.91)^a^	0.19
Self-Directedness	54.93 (22.43)^a^	66.05 (17.64)^b^	71.89 (14.98)^c^	11.48*
Cooperativeness	81.42 (13.21)^a^	87.84 (10.17)^b^	85.58 (9.46)^a, b^	6.06*
Self-Transcendence	36.58 (23.03)^a^	35.24 (19.56)^a^	37.13 (26.95)^a^	0.11
*Eating-related*				
Binge eating	29.00 (7.40)^a^	21.05 (7.71)^b^	17.87 (8.15)^c^	33.01*
Food craving	62.79 (9.52)^a^	55.28 (8.84)^b^	48.95 (15.7)^c^	22.05*
Food addiction	6.77 (2.97)^a^	4.75 (2.99)^b^	3.77 (3.02)^b^	15.20*

Means with different subscripts within a row are significantly different from one another (*p* <.05)

## Discussion

The aim of this study was to characterize patients with overweight or obesity seeking help for compulsive eating using three maladaptive eating behaviors and to compare them on psychological and eating-related variables. A primary care sample was used to represent the heterogeneous portray of patients seeking help for compulsive eating and weight concerns. Three profiles were found namely *Highly disinhibited*,* Moderate sensitivity to eating cues* and *Perceived control over food.* They showed clinically relevant differences regarding psychological and eating-related variables that suggest possible personalized treatment targets.

Profile 1, *Highly disinhibited*, was characterized by the highest scores of disinhibition and susceptibility to hunger and the lowest score of restraint. It also had the highest amount of patient with binge eating disorder. Most patients in this profile meet the diagnostic severity criteria for the disorder. Notably, they experience more frequent and intense binge episodes, as well as impairments in overall functioning [20]. This profile is like the “struggling with food” profile found by Romain et al. [16] in a sample of 126 individuals with severe obesity seeking weight loss, which was characterized by high scores on compulsive eating behaviors like uncontrolled eating and emotional eating as well as low score on restraint. Compared to patients in the two other profiles, those in *Highly disinhibited* showed more severe scores on the impulsivity and depressive symptoms scales, in addition to having the lowest body esteem related to appearance and the youngest age. Patients included in this profile showed the lowest scores for the personality traits of self-directedness and cooperativeness, meaning they are less likely to be able to maintain goal-oriented actions and probably, less likely to adapt properly to change [46]. Adaptation difficulties may result in inappropriate behaviors such as maladaptive eating to cope with daily challenges [47]. Moreover, they showed the most severe scores on all eating-related variables such as binge eating, food craving, and food addiction. These results are consistent with those of Jiménez-Murcia et al. [17] who found, among their three profiles, a “dysfunctional profile” characterized by a higher severity in eating behaviors, a more severe psychological profile (i.e., more impulsivity, less self-directedness, and less cooperativeness) and a younger age. The similarity in maladaptive eating behaviors between our profile and the one identified by Jimenez-Murcia et al. [17] further supports the high comorbidity observed between binge eating and food addiction. These similarities with the *Highly disinhibited* profile support the idea that these pathological psychological characteristics could act as maintaining factors of maladaptive eating behaviors that should be addressed during treatment [48].

Profile 2, *Moderate sensitivity to eating cues*, showed high scores for disinhibition and restraint subscales as well as a moderate level of susceptibility to hunger. This suggests that patients in this profile are less prone to be influenced by perceived internal (e.g., feeling of emptiness) and external (e.g., palatable food publicity) cues related to appetite [1, 22]. It might act as a protective factor against more compulsive eating behaviors, because individuals may feel they are better connected with their hunger and satiety signals. In support of this hypothesis, patients in this profile are not those with the more severe score for binge eating, food craving, and food addiction. Moreover, they were more satisfied with their appearance andhad the greatest sense of cooperativeness, as well as being the oldest of all three profiles. Although the age differences between the profiles are minimal, it could suggest that patients in this profile managed to maintain a better body esteem and lower distress leading them to seek help later. Furthermore, *Moderate sensitivity to eating cues* does not resemble any profiles found in earlier studies. This study is the first to use susceptibility to hunger to classify patients with compulsive eating, which is the variable that mostly distinguishes the profiles. It highlights the relevance of considering how patients perceived their appetite cues and the role susceptibility to hunger may play in their overall eating habits.

Profile 3, *Perceived control over food*, had the highest score of restraint, the lowest score of disinhibition, and by far the lowest score of susceptibility to hunger. Considering that patients in this profile still have a high score of disinhibition when compared to patients with severe obesity and people in the general population [49, 50], it could be hypothesized that they are more likely to control their eating behaviors with restraint to avoid succumbing to their drive towards food. Restraint might be an inefficient strategy to compensate disinhibited eating, resulting in greater alternation between disinhibition and restriction [22]. This eating pattern has been described previously in the literature as a mechanism that may lead to overeating despite that patients may be aiming to eat less by dieting [10, 12]. When looking at psychological variable differences, this profile showed significantly lower scores of impulsivity and depressive symptoms while having the highest score of self-directedness (i.e., being more goal-oriented). It could be hypothesized that the presence of restraint in this profile might give patients an impression of control. Individuals may feel self-determined and in control over their eating habits, which give them the impression of achieving a positive goal [51]. This could explain why patients in this profile show significantly lower scores on several compulsive eating behaviors (i.e., disinhibition, susceptibility to hunger, binge eating, and food craving) and psychological variables (i.e., depressive symptoms and impulsivity).

According to the National Institute for Health and Care Excellence [52], recommendations for patients with obesity seeking help for compulsive eating emphasize the importance of psychotherapeutic intervention before attempting weight loss. However, previous profiling studies have focused on samples of patients with severe obesity awaiting bariatric surgery or seeking weight loss treatment [13–16]. The only study conducted with a clinical sample seeking psychological help for disordered eating excluded patients who did not report food addiction, which makes their recommendations difficult to generalize in primary care [17]. Additionally, their recommendations (e.g., targeting personality traits first, targeting the reward related neural processes, or nutritional changes) are resource-intensive, making them difficult to implement. As a result, no study has yet proposed clinical recommendations that could guide psychotherapeutic interventions for all patients seeking psychological help for obesity and compulsive eating.

Wiss and Brewerton [53] proposed a dimensional model for ED treatment including food addiction. Named *Disordered Eating Food Addiction Nutrition Guide (DEFANG)*, the model aimed to recommend interventions depending on the nature of the eating problem inspired by substance abuse treatment recommendations [53]. The authors argued that some ED are “about the food” when there are high levels of food addiction, lost of control, and overeating. In psychotherapy, they recommend adopting a stricter eating framework to help stabilize food-related impulsivity. One objective may be to steer the patient away from foods that trigger compulsive eating behaviors. Inversely, patients with food and weight-related fears, along with restraint eating patterns, might exhibit ED that are “not about the food”. In such cases, it may be beneficial to expose patients to the foods they fear to restore balance, while also addressing the psychological factors underlying their maladaptive eating behaviors and beliefs about food [53].

Based on the DEFANG model [53], specific interventions may be prioritized depending on the main characteristics of each profile. For instance, *Highly disinhibited* is a profile found recurrently among patients with obesity [15–17]. With high levels of disinhibition, susceptibility to hunger, binge eating, food craving, food addiction, and impulsivity, this profile could benefit from interventions aimed at reducing compulsive eating. A similar treatment approach might also be appropriate for *Moderate sensitivity to eating cues*, though applied with more flexibility. Finally, a possible use of restraint to compensate compulsive eating was identified in *Perceived control over food*. In this case, the “not about the food” eating pathology might point towards a more democratized approach, where prohibitions are reduced, and patients are exposed to a variety of foods [53]. Although the “not about the food” described in the DEFANG model refers to people with anorexia nervosa who are underweight, and from whom the level of restraint is suspected to be much higher, it can still be used to think clinically about patients who exhibit significant restraint, even in the presence of compulsive eating. In addition, psychoeducation on the impact of restraint and the control it gives could help to empower patients who seem stuck in a false sense of self-directedness [51]. These different proposed avenues for intervention reflect the importance of considering characteristics of each profile as it may point in opposite direction for treatment.

This study has limitations to consider. First, the sample was exclusively composed of women. While it allowed to better control for heterogeneity among the sample, it limited generalization. Further studies should replicate the findings using a more diverse sample. Second, BMI was not reported by all patients and the emotion regulation measure was introduced during the study resulting in missing data. This may have reduced the statistical power. Third, the psychiatric comorbidities were not assessed, which makes it impossible to consider their impact on the profiles found. Fourth, having an intuitive eating measure could have helped us better comprehend how susceptibility to hunger differentiates patients in our study [54]. Finally, this study was not preregistered.

## Conclusions

This study identified three profiles, namely *Highly disinhibited*,* Moderate sensitivity to eating cues,* and *Perceived control over food* among a clinical sample of patients with overweight or obesity seeking help for compulsive eating. Significant differences on psychological (i.e., impulsivity, body esteem, depressive symptoms, and personality traits) and eating-related (i.e., binge eating, food craving, and food addiction) variables were found between the profiles. This study highlights mechanisms that seem to prevail in different eating behaviors profiles, which offer intervention targets that should be prioritized when offering psychotherapeutic treatment. Treatment targets could be adapted according to the DEFANG model [53], meaning that patients in the *Highly disinhibited* and *Moderate sensitivity to eating cues* profiles might benefit from an “about the food” treatment approach while those in the *Perceived control over food* profile may be advantaged by a “not about the food” treatment approach. To further explore clinical relevance, a next step would be to test if these three profiles could predict treatment dropout, compliance, and success.

## Data Availability

No datasets were generated or analysed during the current study.
